# Retinoic Acid Signal Negatively Regulates Osteo/Odontogenic Differentiation of Dental Pulp Stem Cells

**DOI:** 10.1155/2020/5891783

**Published:** 2020-06-27

**Authors:** Jiangyi Wang, Guoqing Li, Lei Hu, Fei Yan, Bin Zhao, Xiaoshan Wu, Chunmei Zhang, Jinsong Wang, Juan Du, Songlin Wang

**Affiliations:** ^1^Laboratory of Molecular Signaling and Stem Cell Therapy, Beijing Key Laboratory of Tooth Regeneration and Function Reconstruction, Capital Medical University School of Stomatology, Beijing, China; ^2^Molecular Laboratory for Gene Therapy and Tooth Regeneration, Beijing Key Laboratory of Tooth Regeneration and Function Reconstruction, Capital Medical University School of Stomatology, Beijing, China; ^3^Department of Prosthodontics, Capital Medical University School of Stomatology, Beijing, China; ^4^Xiangya Stomatological Hospital and School of Stomatology, Central South University, Changsha, Hunan, China; ^5^Department of Oral and Maxillofacial Surgery, Xiangya Hospital, Central South University, Changsha, China; ^6^Department of Biochemistry and Molecular Biology, Capital Medical University School of Basic Medical Sciences, Beijing, China

## Abstract

Retinoic acid (RA) signal is involved in tooth development and osteogenic differentiation of mesenchymal stem cells (MSCs). Dental pulp stem cells (DPSCs) are one of the useful MSCs in tissue regeneration. However, the function of RA in osteo/odontogenic differentiation of DPSCs remains unclear. Here, we investigated the expression pattern of RA in miniature pig tooth germ and intervened in the RA signal during osteo/odontogenic differentiation of human DPSCs. Deciduous canine (DC) germs of miniature pigs were observed morphologically, and the expression patterns of RA were studied by *in situ* hybridization (ISH). Human DPSCs were isolated and cultured in osteogenic induction medium with or without RA or BMS 493, an inverse agonist of the pan-retinoic acid receptors (pan-RARs). Alkaline phosphatase (ALP) activity assays, alizarin red staining, quantitative calcium analysis, CCK8 assay, osteogenesis-related gene expression, and *in vivo* transplantation were conducted to determine the osteo/odontogenic differentiation potential and proliferation potential of DPSCs. We found that the expression of *RARβ* and *CRABP2* decreased during crown calcification of DCs of miniature pigs. Activation of RA signal *in vitro* inhibited ALP activities and mineralization of human DPSCs and decreased the mRNA expression of *ALP*, *osteocalcin*, *osteopontin*, and a transcription factor, *osterix*. With BMS 493 treatment, the results were opposite. Interference in RA signal decreased the proliferation of DPSCs. *In vivo* transplantation experiments suggested that osteo/odontogenic differentiation potential of DPSCs was enhanced by inversing RA signal. Our results demonstrated that downregulation of RA signal promoted osteo/odontogenic differentiation of DPSCs and indicated a potential target pathway to improve tissue regeneration.

## 1. Introduction

Retinoic acid (RA), the main active derivative of vitamin A, found in embryos and adult vertebrates [1], is essential for embryonic development [2–4] and, like several other molecules, continues to play vital roles after the development is completed [5]. RA signaling is activated when RA binds to cellular retinoic acid-binding protein (CRABP), which translocates RA from the cytoplasm into the nucleus. In the nucleus, heterodimers of nuclear retinoic acid receptors (RARs) and retinoid X receptors (RXRs) recognize RA and regulate transcription by association with retinoic acid response elements (RAREs) in the promoter regions of DNA [6]. Previous studies have shown the dynamic expression patterns of RA-relative signaling molecules in developing tooth [3, 7–9] and reported that RA signals regulated the initiation and formation of dentition at early stages of development [10–12]. An excess amount of RA has negative effects on the maintenance of stem cell niche and enamel formation [13, 14]. RA signaling is also involved in bone metabolism and osteoblast differentiation [15–17]. Interactions have been reported between RA and several molecules from osteo/odontogenic-related pathways, like bone morphogenic protein (BMP) [18], fibroblast growth factor (FGF) [13], and members of the Wnt signaling pathway [16, 19]. However, to the best of our knowledge, the direct role of RA in dentin mineralization and odontoblast differentiation is not yet reported.

As mesenchymal stem cells (MSCs) have key roles in tissue engineering, their sources and the regulation of their differentiation mechanisms in tissue regeneration are active areas of research. Dental pulp stem cells (DPSCs) have been isolated from an adult dental pulp and are characterized by their high proliferation rate, self-renewal capability, and their potential to differentiate into osteoblasts, odontoblasts, adipocytes, etc. [20]. Currently, DPSCs are widely studied as potential seed cells in regeneration for dentin pulp-like complex and periodontal tissue and bone [21–24]. Our previous studies [25–27] have identified the role of DPSCs in functional root and periodontal regeneration. Our recent research observed that, compared to other mesenchymal stem cells, DPSCs have superior resistance to cellular senescence in culture and under an inflammatory environment [28]. All these observations suggested that DPSCs can be a promising source of MSCs for tooth regeneration. Improvement in the differentiation efficacy of DPSCs can greatly facilitate their utility in tissue regeneration.

In this study, we used deciduous canines (DCs) of miniature pigs between late bell stage and calcification stage to study the expression pattern of RA in the dental papilla (DP) during crown calcification. Using human DPSCs, we investigated if RA had the presumed effects in osteo/odontogenic differentiation of human DPSCs. Our results revealed the negative effect of RA, both in crown calcification and in osteo/odontogenic differentiation of DPSCs, and we successfully improved the regeneration of bone-like tissue by inversing the RA signal, a novel method to promote the bone/dentin regeneration.

## 2. Materials and Methods

### 2.1. Animals

Pregnant miniature pigs were obtained from the Animal Science Institute of Chinese Agriculture University. The gestation age was calculated from the day of insemination. Pregnancy was verified through B-type ultrasonography. All procedures acquired approvement from the Animal Care Use Committee of Capital Medical University (Beijing, China) (Permit Number: AEEI-2016-063). Pregnant pigs were anesthetized and sacrificed as previously described [29]. The DCs were harvested on embryonic day 50 (E50) and embryonic day 60 (E60).

### 2.2. *In Situ* Hybridization (ISH)

RNA probe synthesis and nonradioactive *in situ* hybridization were carried out as described previously [30, 31]. The primers used for reverse transcriptase polymerase chain reaction (RT-PCR) are listed in Table 1. Briefly, total RNA was extracted from DC tooth germs from miniature pigs on E50-60. After RT-PCR, the DNA bands of interest were extracted and their DNA sequences were determined. Digoxigenin- (DIG-) labeled RNA probes were synthesized with DIG-UTP and T7 RNA polymerase (10881767001; Roche, Switzerland) and DIG RNA labeling mix solution (11277073910; Roche). For the staining process, slides were deparaffinized and rehydrated completely and then digested with 1 *μ*g/mL proteinase K for 30 min at 37°C. After refixation with 4% paraformaldehyde- (PFA-) PBS, the sections were dehydrated in 25, 50, 75, and 100% ethanol successively before air-drying for 1 hour. Hybridization was performed overnight with diluted probes in an RNase-free incubator at 70°C. In the next day, the sections were rinsed for 3 to 4 hours and incubated with antibodies (alkaline phosphatase-conjugated anti-digoxigenin, Fab fragments) (11093274910; Roche) overnight. Signal detection was performed with the NBT/BCIP substrate (S3771; Promega, Madison, WI).

### 2.3. Cell Cultures

Human tooth tissues were obtained from impacted third molars, under approved guidelines set by the Beijing Stomatological Hospital, Capital Medical University. All procedures were performed with informed consent from patients. The isolation and culture of DPSCs were performed as reported previously [32]. DPSCs at passages 3 to 5 were used in the following experiments. DPSCs were cultured for 3-14 days in osteogenic induction medium containing 100 *μ*M of ascorbic acid, 2 mM of *β*-glycerophosphate, 1.8 mM of KH_2_PO_4_, and 10 nM of dexamethasone. In the experimental groups, to activate the RA signal, RA (Sigma-Aldrich, Santa Louis, USA) was used and, to inhibit RA signal, BMS 493 (Tocris, Cat. No. 3509), an inverse agonist of pan-RARs, was used. For each experiment, RA and BMS 493 stock solutions were initially diluted in DMSO and then in culture medium for 10^−7^ M, 10^−6^ M, and 10^−5^ M according to a previous literature [13, 19, 33].

### 2.4. Alkaline Phosphatase (ALP) Assay and Alizarin Red Staining

After osteogenic induction for 7 days, ALP activity assay was performed using an ALP activity kit following the instruction from the manufacturer's protocol (Sigma-Aldrich). Signal strength was normalized based on protein concentration. After 14 days of induction, mineralization was detected. The cultured DPSCs were fixed with 70% ethanol and stained in 2% Alizarin red (Sigma-Aldrich). To calculate their calcium contents, the above samples were destained with 10% cetylpyridinium chloride before measuring their absorbance at 562 nm on a multiplate reader. Their calcium contents were derived from a standard calcium curve, constructed using calcium dilutions of the same solution. In each group, the final calcium level was normalized to total protein concentration in duplicate plates.

### 2.5. Cell Proliferation Assay

To analyze the effects of RA signal intervention on DPSC proliferation, cell proliferation assay was performed in 96-well plates using cell counting kit-8 (CCK8; Dojindo, Tokyo, Japan). After osteogenic induction for 1, 2, 3, 4, and 5 days, 10% CCK8 reagent was added to each well and incubated at 37°C for 2 h before measuring the optical density (OD) values of the samples at 450 nm on a microplate reader. Cell proliferation capacities were represented by the OD values.

### 2.6. RNA Isolation, RT-PCR, and Real-Time RT-PCR

Total RNA was extracted from DPSCs using TRIzol reagent (Invitrogen). Using 2 *μ*g of RNA, cDNA was synthesized with random hexamers or oligo (dT) and reverse transcriptase according to the manufacturer's protocol (Invitrogen). Real-time RT-PCR reactions were carried out using the QuantiTect SYBR Green PCR kit (Qiagen, Hilden, Germany) and an iCycleriQ Multi-color Real-time RT-PCR Detection System. The primers used for the specific genes are shown in Table 2.

### 2.7. Transplantation in Nude Mice

The present study was performed in accordance with an approved protocol. Eight-week-old female BALB/c nude mice were maintained with free access to water and regular food. Human DPSCs were cultured at the presence or absence of BMS 493 for 3 days before combining with 40 mg of hydroxyapatite/tricalcium phosphate (HA/TCP) ceramic particles. The mixture was then transplanted subcutaneously on the dorsal side of the nude mice. After 8 weeks, the transplants were harvested and fixed with 10% formalin for 48 h and decalcified in buffered 10% EDTA (pH 8.0) for a month prior to embedding in paraffin wax and sectioning.

### 2.8. Histological Analyses

Sections were stained with hematoxylin and eosin (HE) to detect morphological changes in the DP of DC from miniature pigs and new bone formation in transplants. Masson's trichrome staining was applied to evaluate the collagen fibril deposits. Image-Pro Plus 6.0 (Media Cybernetics, Rockville, MD) was used for qualitative measurement of mineralization.

### 2.9. Immunohistochemistry Staining

Immunohistochemistry staining was carried out as previously described [34]. Briefly, sections were deparaffinized, hydrated, and immersed in 10% H_2_O_2_ for 10 min to quench the endogenous peroxidase. They were incubated with a primary antibody at 4°C overnight. The primary antibodies used here included those against dentin sialophosphoprotein (DSPP) (Cat. No. ab216892, Abcam, Cambridge, UK), osteocalcin (OCN) (Cat No. ab13418, Abcam, Cambridge, UK), and collagen type I (COL-1) (Cat No. NB600–408, Novus Biological Centennial, USA). In the next day, the sections were washed and incubated in secondary antibody at room temperature. DAB staining was performed with DAB Substrate Kit (Cell Signaling, Danvers, MA, USA) and counterstaining with HE. Image-Pro Plus 6.0 (Media Cybernetics, Rockville, MD) was used for the qualitative measurement of mineralization.

### 2.10. Statistical Analysis

All statistical calculations were carried out using SPSS 13.0 statistical software. Student's *t*-test was performed to determine the statistical significance. *P* value ≤ 0.05 was considered to be significant.

## 3. Results

### 3.1. RA Signaling Decreases during Crown Calcification of Deciduous Canine from Miniature Pig

**Figure 1:**
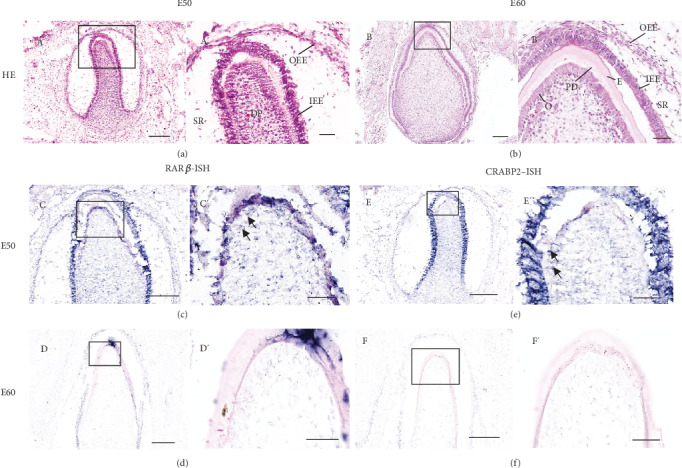
Morphological changes and mRNA expression changes of *RARβ* and *CRABP2* in the DP during crown calcification of DC of miniature pig. (A, A′) In HE staining of DC on E50, tooth germ develops into bell stage; (A′) exhibits the enlarged image of the boxed region in (A). The enamel organ (EO) forms and surrounds the dental papilla (DP) which is located next to the inner enamel epithelium (IEE). (B, B′) In HE staining of DC on E60, tooth germ develops into calcification stage; (B′) exhibits the enlarged image of the boxed region in (B). The elongated odontoblasts (O) appear at the frontier of the DP. The predentin (PD) and enamel are secreted between the DP and EO. The outer enamel epithelium (OEE) and stellate reticulum (SR) can be identified on E50 and E60. The expression patterns of *RARβ* and *CRABP2* in DP are studied by in situ hybridization (ISH); the boxed regions in (C–F) are enlarged in (C′–F′). Expression levels of *RARβ* and *CRABP2* are largely decreased in DP from E50 to E60. Scale bars represent 200 *μ*m (A–F) and 50 *μ*m (A′–F′).

First, we used the DCs from miniature pigs as a model for crown calcification in tooth development. HE staining showed that the enamel of DCs became obvious on E50 (Figures 1(A) and 1(A′)), but in DP, the elongated odontoblasts with secreted predentin did not appear until E60 (Figures 1(B) and 1(B′)). As a result, we designate the development stages of DCs of miniature pigs on E50 and E60 as late bell and calcification stages, respectively. To investigate the role of RA signaling during crown calcification, we studied the RNA expression patterns of *RA receptors α* (*RARα*) and *β* (*RARβ*) and *cellular retinoic acid-binding proteins 1* and *2* (*CRABP1* and *CRABP2*) in DCs from E50 to E60. We found that *RARα* was not expressed in the whole tooth germ of DCs from E50 to E60 and *CRABP1* expression showed little difference between the two stages (data not shown), while the mRNA expressions of *RARβ* (Figures 1(C), 1(C′), 1(D), and 1(D′)) and *CRABP2* (Figures 1(E), 1(E′), 1(F), and 1(F′)) were prominent at E50 in mesenchymal cells and significantly decreased at E60. Considering the simultaneous decrease of *RARβ* and *CRABP2* can downregulate the activation of RA signal, we suggest that RA signal plays a negative role in crown calcification and odontoblast differentiation.

### 3.2. RA Inhibits Osteogenic Differentiation and Cell Proliferation of DPSCs *In Vitro*

**Figure 2:**
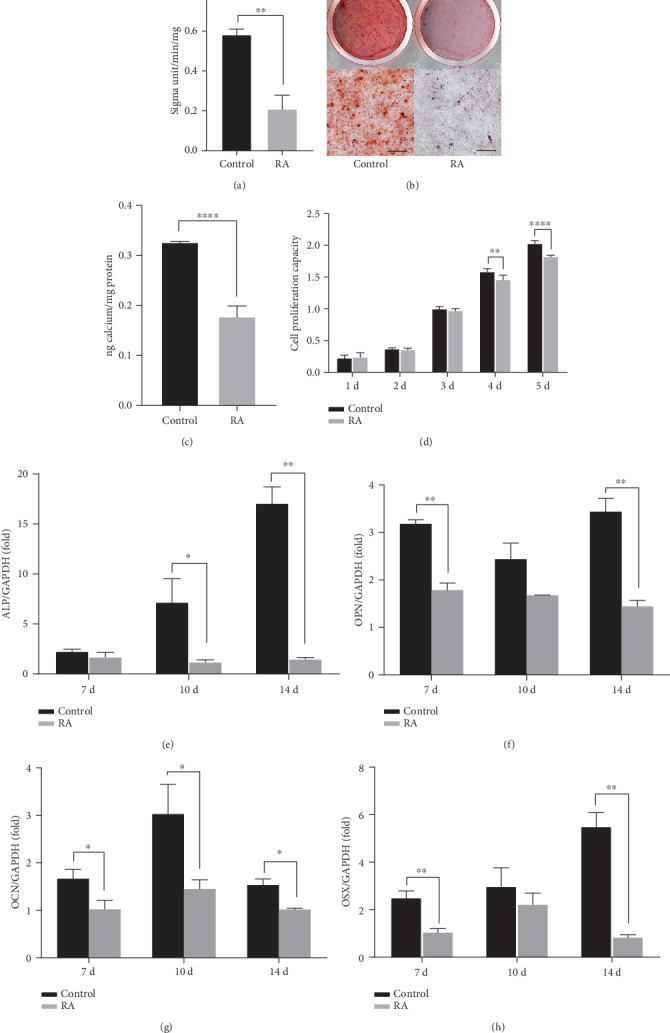
RA inhibits osteo/odontogenic differentiation and proliferation of human DPSCs. (a) ALP activity assay results show RA inhibits ALP activities in human DPSCs after osteogenic induction for 7 days. Alizarin red staining (b) and quantitative calcium measurement (c) results show RA reduces osteogenic differentiation of human DPSCs after osteogenic induction for 14 days. Calcium nodules are observed under a microscope and displayed below (b). (d) CCK8 assays exhibit significant induction in the proliferation of DPSCs by RA after 4-day induction. Real-time RT-PCR results show RA downregulates the expressions of osteogenesis-related markers ALP (e), OPN (f), OCN (g), and transcription factor OSX (h) in human DPSCs. GAPDH is applied as the internal control. Student's *t*-test is performed to calculate statistical significance. All error bars mean the SD (*n* = 3). ^∗^*P* < 0.05, ^∗∗^*P* < 0.01, and ^∗∗∗∗^*P* < 0.0001. Scale bars represent 200 *μ*m (b).

DPSCs are dental stem cells isolated from the dental pulp, which is derived from the dental papilla, the mesenchymal compartment, during development. Based on our above finding, we raise a hypothesis that osteogenic potential of DPSCs could be controlled by RA signal, in addition to its involvement in mineralization of dentin during tooth development. To confirm this, DPSCs were cultured in osteogenic medium with or without RA supplement. First, ALP activity, an early marker for osteogenic differentiation, was analyzed. Experimental groups were treated with 10^−7^ M, 10^−6^ M, and 10^−5^ M of RA, based on a previous literature [13, 19]. In DPSCs, ALP activity decreased on day 7 in the above experimental groups. We chose the minimum effective concentration of 10^−7^ M (Figure 2(a)) for further experiments. After 14 days of culture, DPSCs treated with RA showed fewer mineralization nodules (Figure 2(b)) and quantitative measurements revealed lower concentrations of calcium (Figure 2(c)) than in the control group. Proliferation of DPSCs decreased by RA after 4 days of induction, as seen in CCK8 assay (Figure 2(d)). Real-time RT-PCR was performed to evaluate the expression levels of osteogenic genes including *ALP*, *osteocalcin* (*OCN*), *osteopontin* (*OPN*), and a related transcription factor *osterix* (*OSX*). Compared with the control group, there was significant reduction in the expression of the osteogenic markers (*ALP*, *OCN*, and *OPN*) and *OSX* in RA-treated DPSCs on days 3, 7, 10, and 14 (Figures 2(e)–2(h)). These results show that RA inhibits osteogenic differentiation and proliferation of DPSCs *in vitro*.

### 3.3. BMS 493 Promotes Osteogenic Differentiation and Inhibits Proliferation of DPSCs *In Vitro*

**Figure 3:**
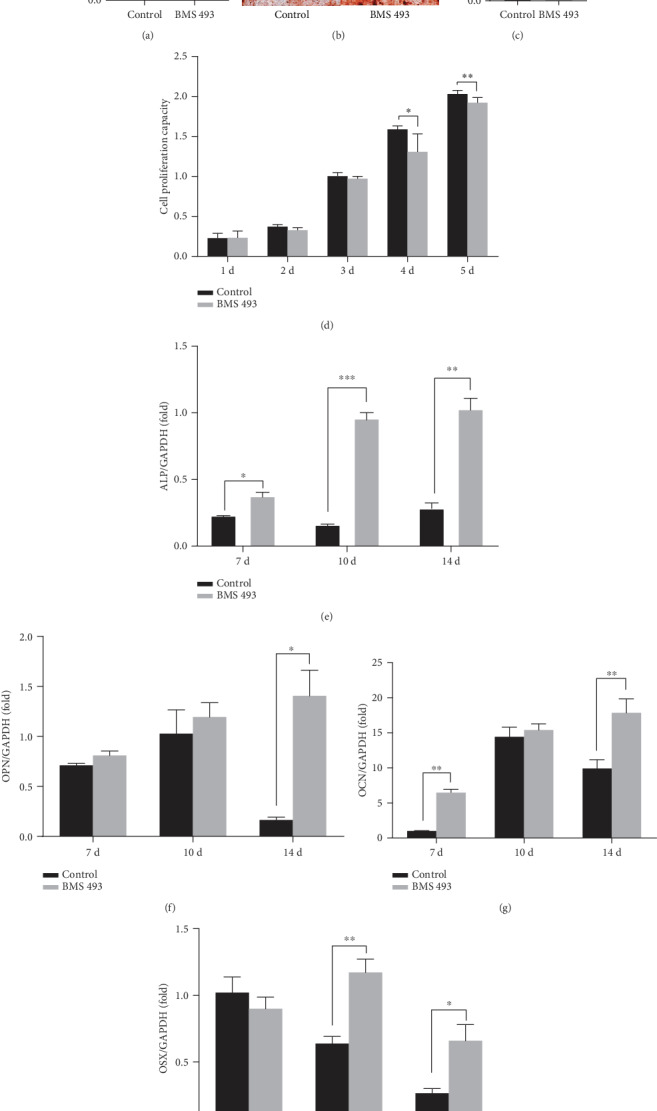
BMS 493 enhances osteo/odontogenic differentiation and inhibits proliferation of human DPSCs. (a) ALP activity assay results exhibit enhanced ALP activities in human DPSCs by BMS 493 after osteogenic induction for 7 days. Alizarin red staining (b) and quantitative calcium measurement (c) results show more mineralization nodules in human DPSCs by BMS 493 after osteogenic induction for 14 days. Calcium nodules are observed under a microscope and displayed below (b). (d) CCK8 assays show the proliferation of DPSCs is largely suppressed by BMS 493 after 4-day induction. Real-time RT-PCR results show BMS 493 upregulates the expressions of osteogenesis-related markers ALP (e), OPN (f), OCN (g), and transcription factor OSX (h) in human DPSCs. GAPDH is applied as the internal control. Student's *t*-test is performed to calculate statistical significance. All error bars mean the SD (*n* = 3). ^∗^*P* < 0.05, ^∗∗^*P* < 0.01, ^∗∗∗^*P* < 0.001, and ^∗∗∗∗^*P* < 0.0001. Scale bars represent 200 *μ*m (b).

To confirm further the role of RA signal in osteogenic differentiation of DPSCs, 10^−7^ M, 10^−6^ M, and 10^−5^ M of BMS 493, an inverse agonist of pan-RARs, were added in the culture medium to modulate the RA signal in DPSCs. With the addition of 10^−7^ M BMS 493, ALP activity increased after 7 days of culture (Figure 3(a)), and more mineralization nodules (Figure 3(b)) and higher concentrations of calcium (Figure 3(c)) were seen. Proliferation of DPSCs decreased in the group treated with 10^−7^ M BMS (Figure 3(d)). Next, we studied the expression of osteogenic genes by semiquantitative RT-PCR. During the 14-day long osteogenic induction, the expression of *ALP*, *OCN*, *OPN*, and *OSX* enhanced significantly in BMS 493-treated DPSCs, compared to the control group (Figures 3(e)–3(h)). Taken together, these results indicate that BMS 493 promotes osteogenic differentiation and inhibits the proliferation of DPSCs *in vitro*.

### 3.4. BMS 493 Promotes Osteogenic Differentiation of DPSCs *In Vivo*

**Figure 4:**
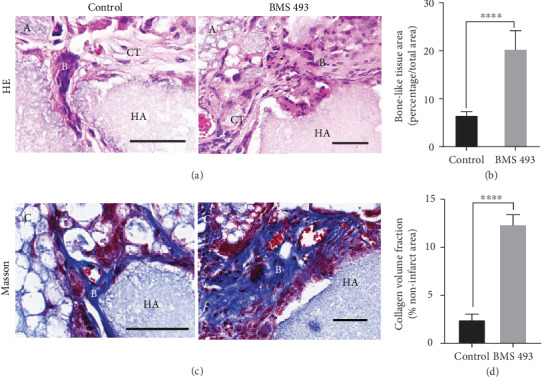
Blocking RA signal enhances the bone-like tissue formation and collagen fibril deposits from human DPSCs in vivo. Human DPSCs cultured at the presence or absence of BMS 493 were transplanted subcutaneously into immunodeficient BALB/c nude mice, and the transplants were harvested after 8 weeks. (A, A′) HE staining of the transplants exhibits improved bone-like tissue formation by BMS 493 treatment. (C, C′) Masson's trichrome staining exhibits more collagen fibril deposits by BMS 493 treatment. Qualitative measurements are used to evaluate bone-like tissue area (B) and collagen volume fraction (D). Scale bars represent 20 *μ*m (A, A′, C, C′). All error bars mean the SD (*n* = 5). ^∗∗∗∗^*P* < 0.0001. B: bone-like tissue; CT: connective tissue; HA: hydroxyapatite/tricalcium carrier.

**Figure 5:**
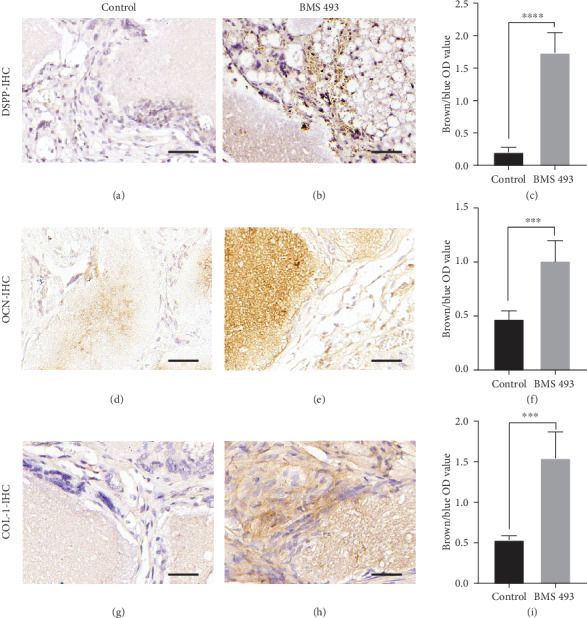
Blocking RA signal enhances the expression of osteo/odontogenic related factors from human DPSCs in vivo. Immunohistochemical (IHC) staining reveals expression levels of DSPP (a, b), OCN (d, e), and COL-1 (g, h). Qualitative measurements of IHC staining show expressions of DSPP (c), OCN (f), and COL-1 (i) are enhanced significantly by BMS 493 treatment. All error bars mean the SD (*n* = 5). ^∗∗∗^*P* < 0.001 and ^∗∗∗∗^*P* < 0.0001.

To test whether the RA signal affects osteogenesis *in vivo*, human DPSCs were treated with or without BMS 493 for 3 days before mixing them with HA/TCP ceramic particles and transplanting subcutaneously into nude mice. After eight weeks, the transplanted tissues were retrieved and decalcified. HE staining showed the formation of more bone-like tissue in BMS 493-treated DPSCs than in the control groups (Figures 4(A) and 4(A′)). In Masson's trichrome staining, collagen fibrils are stained blue, in contrast to a red background of cells and other structure. Here, it exhibited larger areas of blue, indicating more fibrous tissue formation, which is an early stage in bone formation, in BMS 493-treated DPSCs, compared to controls (Figures 4(C) and 4(C′)). Qualitative measurements revealed more newly formed bone tissues (Figure 4(B)) and collagen fibrous tissues (Figure 4(D)) in BMS 493-treated groups than in control groups. Further, IHC was performed to evaluate the osteo/odontogenic marker expressions. Expression levels of DSPP, OCN, and COL-1 were significantly higher in the BMS 493-treated group than in the control (Figures 5(a), 5(b), 5(d), 5(e), 5(g), and 5(h)). Qualitative measurements confirmed the relative differences in the expression of DSPP, OCN, and COL-1 between the BMS 493 group and control group (Figures 5(c), 5(f), and 5(i)). Taken together, these results show that the bone/dentin regenerative potential of DPSCs can be enhanced substantially by inversing RA signal *in vivo*.

## 4. Discussion

Retinoic acid is an early signal in embryonic development and has a crucial role in the early stage of tooth development [10–12]. Studies have shown the participation of RAR signals in postnatal bone metabolism [35]. A negative role of RA and RAR was reported in osteogenesis and bone mineralization [16, 19, 36]. In murine tooth development, RAR*β* expression is initiated during bell stage and is seen in odontoblasts [37]. The expression of CRABP2 is seen in dental mesenchymal cells and decreases during dentin development in mouse [38]. CRABP2 knockdown enhances odontoblastic differentiation of human DPSCs. It is downregulated during osteogenic differentiation from myogenic progenitor cells and negatively regulates osteogenic differentiation [39]. Miniature pigs are similar to humans in their mandibular anatomy and diphyodont dentition [40]. In the present study, the simultaneous decrease in the expression of *RARβ* and *CRABP2* in dental mesenchymal cells of miniature pigs during tooth development indicated the negative role of RA signal in odontogenic differentiation and dentin mineralization.

In recent years, DPSCs are proved to be a promising choice in dentin and bone regeneration. The ability to manipulate the stem cells accurately to a desired cell lineage remains a much pursued goal of research [41]. Given the negative role of RA in mineralization, we propose that blocking RA signal may be an effective method for dentin regeneration from DPSCs. Knocking down the CRABP2 in DPSCs through transfection with lentivirus promoted the odontogenic differentiation of DPSCs *in vitro* [38]. However, procedural difficulties and safety issues are the roadblocks in the clinical application of lentivirus transfection. In this scenario, inversing the RA signal by BMS 493 is a new method to improve the osteo/odontogenic differentiation of DPSCs in tissue regeneration, both *in vitro* and *in vivo*. The present results showed that BMS 493 enhanced the osteo/odontogenic differentiation potential in DPSCs *in vitro* and enhanced the regeneration of bone-like tissue *in vivo*. Although previous studies have shown the RA signal had a significant role in regulating the proliferation of different cell types [42, 43], interesting results were investigated in our work that the proliferation of human DPSCs was decreased whenever activating or blocking RA signal after 4 days of culture, which suggests that the proliferation of DPSCs may be independent from RA signaling and the osteogenic induction may influence the cell proliferation.

**Figure 6:**
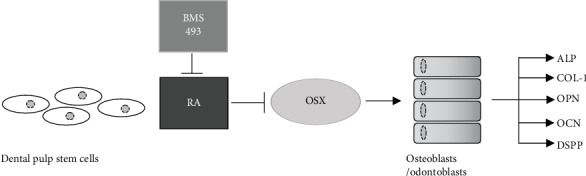
The schematic diagram of the effect of RA on DPSCs. Activation of RA inhibits osteo/odontogenic differentiation of DSPCs and the expression levels of osteo/odontogenic related factors ALP, COL-1, OPN, OCN, and DSPP by downregulating the expression of OSX.

Different stages of the osteo/odontogenic differentiation of MSCs are characterized by several markers. ALP participates in bone mineralization and is an early osteogenic differentiation marker [44, 45], while OPN and OCN are specific matrix proteins associated with bone metabolism and remodeling and are late osteoblastic differentiation markers in MSCs [45, 46]. During osteogenic induction *in vitro*, we studied the expression of *ALP*, *OPN*, and *OCN* by real-time RT-PCR. Their synchronous decrease due to RA treatment indicated the negative effect of RA in osteogenesis of DPSCs in both early and late differentiation periods. OSX is a key transcription factor specifically expressed in developing bones [47] and enhances the osteogenic differentiation potential of stem cells [48, 49]. In mouse tooth development, *OSX* was reported to be highly expressed in osteogenic mesenchyme and odontoblasts, and its overexpression in mouse odontoblast-like cells resulted in enhanced transcription of an odontogenic marker, *DSPP* [50]. By real-time RT-PCR, we detected the downregulation of *OSX* due to RA and its enhancement due to BMS 493. These results suggested a role for OSX in regulating RA signal in osteogenic differentiation of DPSCs (Figure 6). Future research needs to explore the role of RA and related signals during osteo/odontogenic differentiation and focus on the specific mechanisms of action of RA on OSX. DSPP is an odontogenic marker widely expressed in mature odontoblasts and dentin [51, 52]. COL-1 is a major component of mineralized tissues, and reports support its potential to improve the survival and expression of osteogenic and chondrogenic phenotypes in MSCs *in vivo* [53]. The present research proved that treatment with BMS 493 improved the expression of DSPP, COL-1, and OCN as well as enhanced the mineralization of DPSCs *in vivo*.

In contrast, some studies have suggested a positive role for the RA pathway in osteogenic differentiation of MSCs [54–56] and dental pulp cells [57–59]. However, their *in vitro* induction time was mostly shorter than 7 days while osteogenesis is a long and dynamic process. In addition, a long-term *in vitro* culture of stem cells from periodontal ligaments and pulp of human exfoliated deciduous teeth showed improved osteogenic differentiation by RA [60]. Different culture conditions, varied induction time, and different cell types may explain this discrepancy. In any case, the specific mechanisms of RA signal in osteo/odontogenic differentiation remain to be delineated.

## 5. Conclusion

This study indicated that the RA signaling pathway was involved in dental crown calcification and demonstrated that activation of RA signal decreased the osteogenic differentiation potential of DPSCs and blocking RA signal enhanced it *in vitro* and *in vivo*. The modulation of RA signaling has potential for improving the tissue regeneration and explains the mechanisms of osteogenesis and odontogenesis. The relationship between RA and its downstream signals needs to be studied in greater details to understand this process better.

## Figures and Tables

**Table 1:** Primer sequences used in the RT-PCR.

Gene symbol	Primer sequences (5′-3′)
RAR*α*-sense	CTAAACGTCTGCCAGGCTTC
RAR*α*-antisense	CGGGATGCATGAAATGGCTG
CRABP2-sense	CCCAACTTCTCTGGCAACTGG
CRABP2-antisense	TCTAGAAGGAAGGGTAGGGGAG

**Table 2:** Primer sequences used in the real-time PCR.

Gene symbol	Primer sequences (5′-3′)
GAPDH-F	GGAGCGAGATCCCTCCAAAAT
GAPDH-R	GGCTGTTGTCATACTTCTCATGG
ALP-F	GACCTCCTCGGAAGACACTC
ALP-R	TGAAGGGCTTCTTGTCTGTG
OPN-F	CGCAGACCTGACATCCAGTA
OPN-R	GTGGGTTTCAGCACTCTGGT
OCN-F	TCACACTCCTCGCCCTATTG
OCN-R	GGGTCTCTTCACTACCTCGC
OSX-F	CCCACCTCAGGCTATGCTAA
OSX-R	GCCTTGTACCAGGAGCCATA

## Data Availability

All the data used to support the findings of this study are available from the corresponding author upon request.

## Supplementary Materials

Supplementary Materials Supplementary Figure 1: Intervention in RA signal with different concentrations of RA and BMS 493 in vitro alters the ALP activities differentially (A, B).
