# Supplementary material for: Retinoic Acid Signal Negatively Regulates Osteo/Odontogenic Differentiation of Dental Pulp Stem Cells
Source: Stem Cells Int. 2020 Jun 27;2020:5891783. doi: 10.1155/2020/5891783 (PMC7336240; doi:10.1155/2020/5891783)
Supplement: Supplementary Materials — Supplementary Figure 1: Intervention in RA signal with different concentrations of RA and BMS 493 in vitro alters the ALP activities differentially (A, B). [file 5891783.f1.pdf]

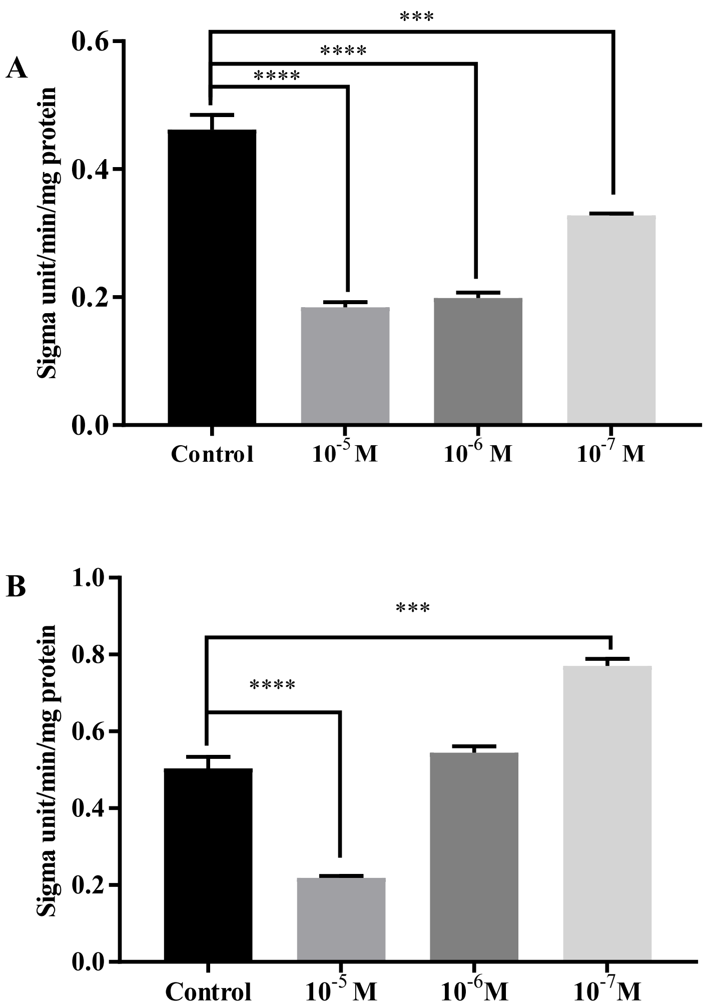

Supplementary Figure 1. Intervention in RA signal with different concentrations of RA and BMS 493 *in vitro* altered the ALP activities differentially (A, B). ALP activities were significantly suppressed by 10^{-7} M, 10^{-6} M, 10^{-5} M RA (A). ALP activities were significantly enhanced by 10^{-7} M BMS 493, but decreased by 10^{-5} M BMS 493 and changed little by 10^{-6} M BMS 493 (B).
